# EEG channel and feature investigation in binary and multiple motor imagery task predictions

**DOI:** 10.3389/fnhum.2024.1525139

**Published:** 2024-12-17

**Authors:** Murside Degirmenci, Yilmaz Kemal Yuce, Matjaž Perc, Yalcin Isler

**Affiliations:** ^1^Kutahya Vocational School, Kutahya Health Sciences University, Kutahya, Türkiye; ^2^Department of Computer Engineering, Alanya Alaaddin Keykubat University, Antalya, Türkiye; ^3^Faculty of Natural Sciences and Mathematics, University of Maribor, Maribor, Slovenia; ^4^Community Healthcare Center Dr. Adolf Drolc Maribor, Maribor, Slovenia; ^5^Complexity Science Hub Vienna, Vienna, Austria; ^6^Department of Physics, Kyung Hee University, Seoul, Republic of Korea; ^7^Department of Biomedical Engineering, Izmir Katip Celebi University, Izmir, Türkiye

**Keywords:** brain-computer interface, electroencephalogram, feature and channel investigation, feature selection, machine learning, motor imagery task classification

## Abstract

**Introduction:**

Motor Imagery (MI) Electroencephalography (EEG) signals are non-stationary and dynamic physiological signals which have low signal-to-noise ratio. Hence, it is difficult to achieve high classification accuracy. Although various machine learning methods have already proven useful to that effect, the use of many features and ineffective EEG channels often leads to a complex structure of classifier algorithms. State-of-the-art studies were interested in improving classification performance with complex feature extraction and classification methods by neglecting detailed EEG channel and feature investigation in predicting MI tasks from EEGs. Here, we investigate the effects of the statistically significant feature selection method on four different feature domains (time-domain, frequency-domain, time-frequency domain, and non-linear domain) and their two different combinations to reduce the number of features and classify MI-EEG features by comparing low-dimensional matrices with well-known machine learning algorithms.

**Methods:**

Our main goal is not to find the best classifier performance but to perform feature and channel investigation in MI task classification. Therefore, the detailed investigation of the effect of EEG channels and features is implemented using a statistically significant feature distribution on 22 EEG channels for each feature set separately. We used the BCI Competition IV Dataset IIa and 288 samples per person. A total of 1,364 MI-EEG features were analyzed in this study. We tested nine distinct classifiers: Decision tree, Discriminant analysis, Logistic regression, Naive Bayes, Support vector machine, k-Nearest neighbor, Ensemble learning, Neural networks, and Kernel approximation.

**Results:**

Among all feature sets considered, classifications performed with non-linear and combined feature sets resulted in a maximum accuracy of 63.04% and 47.36% for binary and multiple MI task predictions, respectively. The ensemble learning classifier achieved the maximum accuracy in almost all feature sets for binary and multiple MI task classifications.

**Discussion:**

Our research thus shows that the statistically significant feature-based feature selection method significantly improves the classification performance with fewer features in almost all feature sets, enabling detailed and effective EEG channel and feature investigation.

## 1 Introduction

Brain-Computer Interface (BCI) is a particularly created system to provide a direct path between the human brain and a computer-aided device. Its main objective is the facilitation of daily activities for specific individuals with severe motor disabilities due to various neurodegenerative disorders like amyotrophic lateral sclerosis, brain stem stroke, spinal cord injury, and various other diseases (Wolpaw, 2002). In the process of a BCI system, the main goal is the determination of the intent of an individual from various electrophysiological signals. Fundamentally, many BCI systems design a 5-stage algorithm, the stages of which are data acquisition, data preprocessing, feature extraction, feature selection, classification and performance evaluation (Isler, 2009; Degirmenci et al., 2023).

The first stage of such systems is called data acquisition. At this stage, data representing brain activities are collected. Various neuroimaging techniques such as Electroencephalography (EEG), functional Magnetic Resonance Imaging (fMRI), Magnetoencephalography (MEG), Positron Emission Tomography (PET), and optical imaging are available to collect brain activity in the literature (Sayilgan et al., 2022). However, EEG is the most popular technique among the different modalities due to its various advantages, especially ease of use, cheaper equipment, non-invasiveness, reliability, and disposability. Therefore, EEG signals have been mainly used to detect brain activities for BCI research studies (Chen et al., 2019; Sayilgan et al., 2022).

In prosthetic device design, various challenges were addressed in the literature. Designing reliable, functional, robust, and cost-effective BCIs as prosthetic systems is crucial for user acceptance. Many EEG-based BCI systems were designed to process various control signals (Wang et al., 2006; Sayilgan et al., 2022) and enable paralyzed patients to control a prosthetic device. Among these control signals, Motor Imagery (MI) is a well-structured methodology for BCI control because it induces more patterns of Event-Related Desynchronization/Synchronization (ERD/ERS) in various frequency bands. The distinctive patterns in MI-EEG signals enable the differentiation of multiple MI tasks. Consequently, the MI-EEG signal classification has become a major focus in BCI research.

In recent decades, various pattern recognition methods have been proposed to identify specific patterns within EEG-based signals for MI tasks. Machine learning, as one of those methods defines pattern recognition as a process consisting of the following stages: data collection, feature extraction, feature selection, and classification. In this respect, various feature extraction, feature selection, and machine learning algorithm methods have been used to analyze EEG signals following the data acquisition stage. Feature extraction, channel selection, and feature selection methods act as significant sub-components of MI-based BCI systems (Bashashati et al., 2007).

The feature extraction stage, which is the first crucial sub-component, determines a set of EEG signal features for effective discrimination of multiple MI tasks. The extracted features for MI-EEG signals analysis can be divided into the following categories: (i) time-domain features such as such as mean, mean absolute value, variance and the Hjorth parameters (Vidaurre et al., 2009; Sayilgan et al., 2021a), (ii) frequency-domain features such as the frequency of maximum spectral power, and the signal power within an extracted feature band using Fourier Transform (FT) and Power Spectral Density (PSD) (Mensh et al., 2004; Djamal et al., 2017), (iii) time-frequency domain features using various time-frequency representation algorithms such as Short-Time Fourier Transform (STFT) and Wavelet Transform (WT) (Ha and Jeong, 2019; Chaudhary et al., 2020), (iv) spatial features such as Common Spatial Patterns (CSP) and their different types, (Blanco-Diaz et al., 2022; Ang et al., 2012; Samek et al., 2013; Wu et al., 2014), and (v) various transformation-based features such as Empirical Mode Decomposition (EMD) and its various versions, and Intrinsic Time-Scale Decomposition (ITD) (Mwata-Velu et al., 2021; Degirmenci et al., 2024).

The second stage is called feature selection. The goal of feature selection is to avoid the curse of dimensionality and enable the design of cost-effective BCI systems. Feature selection methods have been used to extract the most informative and discriminative features and improve classifier performance for MI-based BCI systems. Among the various feature selection methods, Genetic Algorithms (GA) and Principal Component Analysis (PCA) have substantially been employed and implemented in the development of BCI systems (Bashashati et al., 2007; Mousa et al., 2016). Besides these methods, several other feature selection methods have also been used in BCI systems (Isler, 2009; Degirmenci et al., 2023; Yesilkaya et al., 2023).

In the aforementioned MI task classification studies, various pattern recognition methods have produced successful results. Consequently, various feature extraction, feature selection, and classification algorithms have been proposed for recognizing patterns in EEG signals. The literature studies show that it is important to utilize a combination of different types of feature extraction methods, feature selection methods, and machine learning algorithms and investigate them to design an efficient and cost-effective MI-based BCI system. Exploring the most effective combination of these methods through performance comparisons is the subtle aspect as it is crucial for the same purpose. The detailed comparison of different feature extraction methods together with different combinations of feature selection methods and different machine learning algorithms is relevant to explore effective combination of these methods. Additionally, studying the impact of the channel selection process is an important analysis for MI-based BCI systems. It is thought that, the analysis of effectiveness of different features from different feature categories and the effectiveness of different EEG channels will greatly contribute to this research area.

### 1.1 Related works

Recent studies have proposed or investigated various EEG features, feature selection methods, and machine learning algorithms to analyze MI-EEG signals.

Verma et al. (2014) conducted a study in 2014 by evaluating Discrete Wavelet Transform (DWT) and cross-correlation based features and implementing various classification algorithms to find the best feature extraction method and classification algorithm. They used five different machine learning algorithms to classify their feature sets. They achieved an average accuracy of 99.40% using DWT and Least-Square Support Vector Machine (LS-SVM) algorithm for binary-class extremity movement task classification on Dataset IVa of BCI competition III.

Lotte et al. (2009) presented subject-independent and subject-dependent BCI design that proposed a binary-class extremity movement task classification using the BCI Competition IV dataset IIa. They used the Filter Bank Common Spatial Pattern (FBCSP) algorithm by providing multi-resolution frequency decomposition and linear classification algorithms. Using these methods, they achieved the highest accuracy of 70.99% and 81.56% for the subject-independent and subject-dependent BCI design, respectively.

In 2020, Tabar and Halici (2016) proposed a deep learning-based approach using BCI Competition IV dataset 2b for binary-class extremity movement task classification. EEG signals were converted into 2D time-frequency maps using STFT. These feature maps were passed as inputs to Convolutional Neural Networks (CNN) architecture. They concluded that their proposed methods achieved a high accuracy of 77.60% for binary classification.

In 2017, Djamal et al. (2017) recommended a binary-class extremity movement task classification study using Fast Fourier Transform (FFT) and Learning Vector Quantization Network (LVQN). They only used one EEG channel (FP1 EEG channel) for EEG signal acquisition and signal processing. They obtained accuracy value of 70.00% with the FFT-based method.

In 2020, Molla et al. (2020) performed binary-class extremity movement task classification using the CSP method and Neighborhood Component Analysis (NCA)-based feature selection method. Their proposed methods using the Support Vector Machine (SVM) algorithm achieved average accuracy of 81.52% for subject-dependent binary classification on BCI Competition IV dataset 2b.

In 2023, Kabir et al. (2023) proposed binary-class extremity movement classification using CSP method for feature extraction stage. On the other hand, they examined the effects of different feature selection methods such as Correlation-based Feature Selection (CFS), Minimum Redundancy and Maximum Relevance (mRMR), and multi-Subspace Randomization and Collaboration-based unsupervised Feature Selection (SRCFS). They demonstrated the superiority of their proposed methods by using the SRCFS method and the Linear Discriminant Analysis (LDA) algorithm.

Gaur et al. (2015) presented an EMD-based approach to classify two different extremity movement tasks from BCI Competition IV dataset IIa. They selected and used only three channels, namely C3, C4, and/or Cz channels, for their proposed methods. Using these selected channels and the EMD algorithm, they achieved an average success of 70.20% with the LDA algorithm.

Mohamed et al. (2018) conducted a four-stage extremity movement task classification study using the ITD algorithm and Artificial Neural Networks (ANNs) algorithm. Using their proposed method, they achieved an average success of 92.20%.

In 2020, Dong et al. (2020) proposed a multi-class extremity movement task classification study using a novel hybrid kernel function relevance vector machine that combined the Gaussian kernel function and the polynomial kernel function. They used Phase Space Reconstruction (PSR) to project EEG data from the time domain into high-dimensional phase space. Then, they applied “One vs. One” Common Spatial Pattern (OVO-CSP) method to evaluate the characteristics of the Phase Space Common Spatial Pattern (PSCSP) features. These features were evaluated with their proposed hybrid structure. They achieved an average accuracy of 74.39% using Independent Component Analysis (ICA), PSR, and CSP methods on BCI Competition IV dataset IIa.

In 2024, Amiri et al. (2024) proposed channel selection using deep learning for multi-class extremity movement task classification. They used a flat CNN architecture for their feature extraction, channel selection, and classification process. They compared their channel selection method with different feature selection methods. According to their experimental results, they achieved 72.01% accuracy with their proposed methods and this accuracy value was higher than the success of other studies based on channel.

As outlined above, there exist many pattern recognition methods that were applied for the classification of extremity movement task. Majority of the proposed studies implement a complex classification algorithm that is unsuccessfully combined with all extracted features or selected features. The methods used generate a computational effort during classification and the classification performance remains at low values despite the load. Because these studies were not planned with detailed feature and effective channel analyses, on the contrary, they were designed and conducted to test the classification performance with more complex approaches (e.g., deep learning-based feature extraction and classification methods, and time-frequency representation based feature extraction methods).

To date, the effect of channel selection together with feature selection have not been investigated in literature. In this study, we used various MI-EEG features from different feature domains, such as time-domain, frequency domain, time-frequency domain, and non-linear domain of EEG signals, to analyze the success of feature extraction methods comparatively. We investigated four different feature sets and their two different combination feature sets. Additionally, we investigated the effects of the statistical significance-based feature selection method on each extracted feature set separately. One of the most important steps of this study is that it is the first study to apply detailed features and channel activity analysis, which also provides a different and new perspective on MI task classification studies. Feature and channel analysis were performed using statistically significant feature distribution, which represent the selected statistically significant feature distribution among 22 EEG channels for each feature set (time-domain, frequency-domain, time-frequency domain, and non-linear domain). We used nine different classification algorithms to reveal the effect of these methods. The classification was carried out using both all features and selected features to investigate the effect of the statistically significant-based feature selection method in this study.

### 1.2 Contributions

The primary contributions of this study are as follows:

- Various feature domains, including time, frequency, time-frequency, and non-linear domains, were implemented to the feature extraction process, and the effects of these feature sets were investigated for binary-class and multi-class extremity movement task classifications separately.
- The effect of the statistical significance-based feature selection method was investigated for each feature set on binary-class and multi-class extremity movement task classifications.
- Feature and channel analysis were implemented using channel-based statistically significant feature distribution among 22 EEG channels for binary-class and multi-class extremity movement task classifications.
- To show the effect of the statistically significant feature selection method we comparatively evaluated nine different classifier algorithms using six different feature sets of all features.
  Finally, it must also be noted that this is the **first study** where the detailed feature and channel analysis are implemented for each feature set in addition to the investigation of the effects of different feature domains on extremity movement task classification, to the best of our knowledge.

## 2 Materials and methods

**Figure 1:**
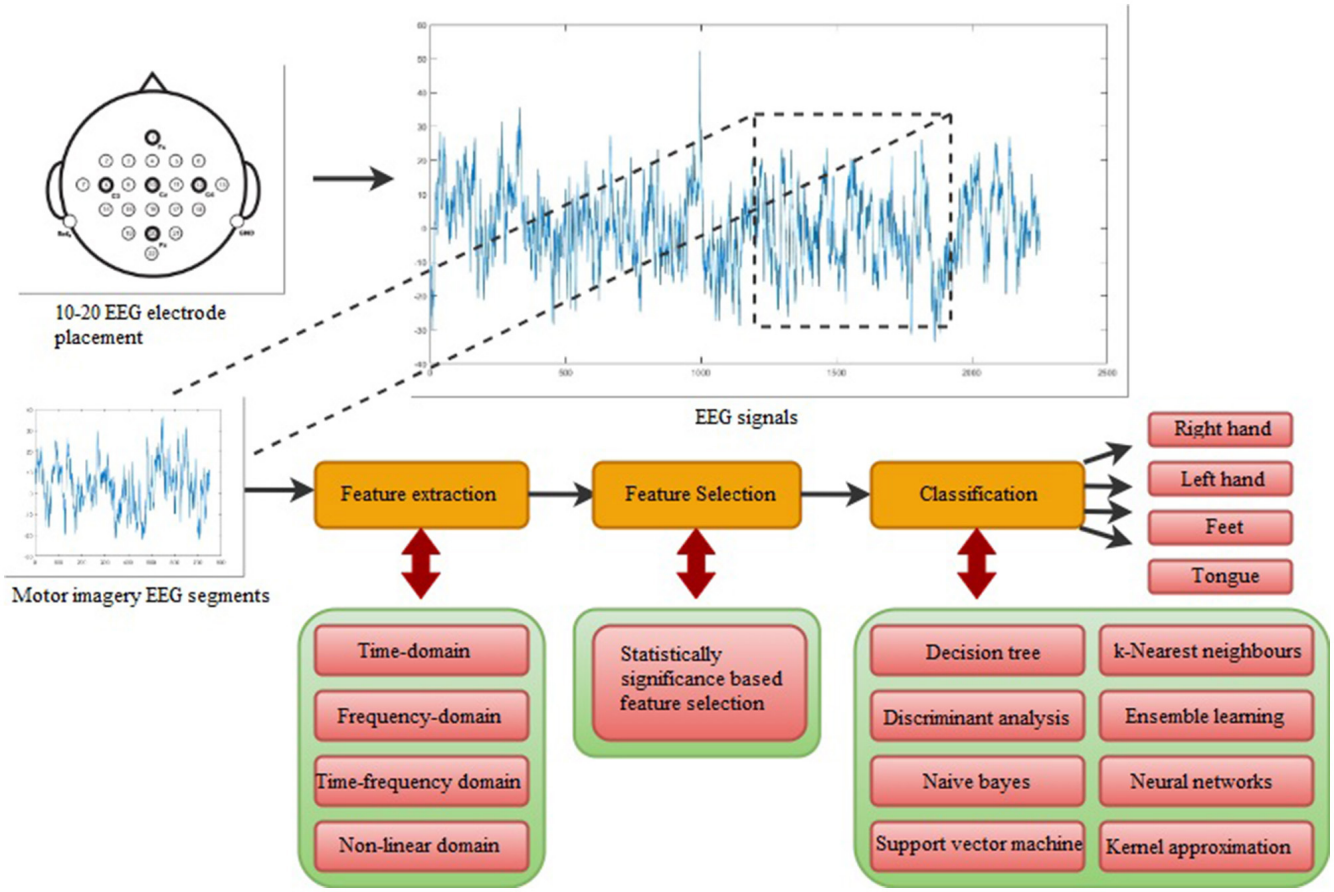
The flowchart of the suggested multi-class extremity movement classification study.

The design of this study includes five stages, which were performed in the given order: data acquisition, feature extraction, feature selection, classification, and performance evaluation. The detailed background regarding each stage is provided in the respective sub-headings. The visual workflow of the multi-class extremity movement classification study is shown in Figure 1 with its five stages. Furthermore, in this study, motor imagery tasks of the right and left hands were differentiated for binary-class extremity movement classification.

### 2.1 Data acquisition and dataset

As the source of data, BCI Competition IV Dataset IIa, which is a publicly available dataset for motor imagery EEG signal analysis, is adopted for binary and multiple extremity movement task classifications (Brunner et al., 2008). It consists of 22-channel EEG data that were recorded from 9 subjects (4 women and 5 men). The sampling rate was set at 250 Hz and EEG data were recorded via 22 Ag/AgCl electrodes. While recording of EEG signals, two filters were applied, i.e., a band pass filter operating between 0.5 Hz and 100 Hz and an additional 50 Hz notch filter, which is used to suppress line noise (Yan et al., 2021). Acquisition of EEG signals occurred during 4 MI tasks. Therefore, this dataset includes data for 4 different MI tasks, which are the imagination of movement of the left hand, right hand, feet, and tongue. The experiment is scheduled as two different sessions, each of which include six runs executed in two days. There are 12 trials available for each motor imagery task in a run, giving a total of 48 trials available for each run. Moreover, 288 trials were conducted after 6 runs for each subject. Therefore, cue-based motor imagery signals were recorded by imagining movements of four different extremity. The corresponding 3 s motor imagery EEG signals were segmented from long EEG signals for each trial before feature extraction and in further stages.

### 2.2 Feature extraction

Within the scope of this study, time-domain, frequency-domain, time-frequency domain, and non-linear features were calculated by utilizing 22-channel EEG signals to provide feature sets after extraction of MI EEG segments.

- **Time-domain feature set:** In the time-domain feature set, 24 different time-domain features were extracted using the directly original field of the EEG signals. These time-domain features are based on the amplitude and statistical changes of the EEG signal (Yesilkaya et al., 2023; Degirmenci et al., 2023).
- **Frequency-domain feature set:** The frequency-domain feature set was created using FT. Frequency-domain features were extracted based on the frequency-domain representation of the MI-EEG signal. The different EEG sub-bands, which are delta (δ), theta (θ), alpha (α), beta (β), and gamma (γ), were embedded in frequency-domain of original EEG signals. These EEG sub-bands were extracted from frequency representation of EEG segments by utilizing the Fast Fourier transform. Following the extraction of EEG sub-bands, the relevant and distinctive MI frequency characteristics were computed based on the energy, variance, and entropy values of different EEG sub-bands for each EEG segment. These frequency-domain features provide information about how power, variance, and entropy (irregularity) change in definite corresponding frequency bands. Their mathematical evaluations are detailed as follows (Sayilgan et al., 2021a; Degirmenci et al., 2023):

(1) $$\begin{array}{l} Energy_{f}=\overset{M}{\underset{k=1}{\sum }}y{\left(k\right)}^{2} \end{array}$$

(2) $$\begin{array}{l} Variance_{f}=\frac{1}{M-1}\cdot \overset{M}{\underset{k=1}{\sum }}{\left(y_{k}-\bar{y}\right)}^{2} \end{array}$$

(3) $$Entropy_{f}=\frac{1}{log(M)}\cdot \sum_{k=1}^{M}P(y(k))log(P(y(k))$$

where *f* denotes the type of EEG sub-bands, *M* denotes the maximum frequency, and *y*(*k*) denotes the FT of a real discrete time EEG segment. In the formula, “$\bar{y}$” indicates the average of the “y” signal. The probability of the EEG segment, which is in the corresponding frequency band, is denoted as *P*(*y*(*i*)).

- **Time-frequency domain feature set:** A time-frequency domain feature set was obtained using Wavelet Transform (WT). Using this algorithm, EEG signals were divided into frequency bands (δ, θ, α, β, and γ). Then, these frequency bands' energy, variance, and entropy values were calculated as time-frequency domain features. DWT utilizes both time and frequency domain information of EEG signals, and its several filters and bandwidths provide multi-resolution analysis (Sayilgan et al., 2021a). It can be utilized as a dual Finite-Impulse Response filter. Utilizing the frequency responses of these filters, high-frequency and low-frequency components of EEG signals are decomposed from EEG signals. The identical wavelet coefficients are selected in both Low-Pass (LP) and High-Pass (HP) filters for the multi-resolution algorithm of WT (Gandhi et al., 2011). The scaling parameter, defining the oscillatory frequency and the length of the wavelet, is associated with the coefficients of LP filter, while the wavelet function is associated with the coefficients of HP filter. The outputs of these filters are indicated as the approximate *(a)* coefficients and the detailed *(d)* coefficients, respectively. The original EEG time series are completely decomposed as *(a)* and *(d)* coefficients based on the defined decomposition level. The subsets of the corresponding coefficients of decomposition levels are included depending on the frequency domain of EEG sub-bands for the decomposition of five EEG frequency bands. In the extraction of the time-frequency domain feature set, the Wavelet packet decomposition is utilized, and the decomposition of the EEG frequency bands was performed at seven decomposition level for 250 Hz sampling frequency of EEG time series (Degirmenci et al., 2023). The “Haar” wavelet function is selected for Wavelet packet decomposition application in the feature extraction process (Sayilgan et al., 2021a; Degirmenci et al., 2023). Following the execution of this algorithm, five different EEG sub-bands were extracted from EEG signals and their energy, variance, and entropy values evaluated as features. According to the following mathematical formulations, the energy of each decomposition level was evaluated (Gandhi et al., 2011):

(4) $$\begin{array}{l} Energy_{d_{i}}=\sum_{j=1}^{N}{\left|d_{ij}\right|}^{2},i=1,2,3,...,l \end{array}$$

(5) $$Energy_{a_{i}}=\sum_{j=1}^{N}{\left|a_{ij}\right|}^{2},i=1,2,3,...,l$$

where the detail (*d*__*i*_*j*_) and approximate (*a*__*i*_*j*_) coefficients indicate the corresponding subsets for each frequency band. The wavelet decomposition level, which is defined in [1, *l*], is indicated as *i = 1,2,3, ...,l*. *N* represents the number of *d* and *a* coefficients.

The mathematical formula for computing variance of each decomposition level is defined as follows (Gandhi et al., 2011):


(6) $$\begin{array}{l} \;Variance_{i}=\frac{1}{N-1}\cdot \sum_{j=1}^{N}{\left(d_{ij}-\mu_{i}\right)}^{2},i=1,2,3,...,l\\ \mu_{i}=\frac{1}{N}\cdot \sum_{j=1}^{N}d_{ij},i=1,2,3,...,l \end{array}$$

where μ_*i*_ denotes the mean of the decomposition level.

The entropy of each decomposition level is evaluated based on the following equation (Isler, 2009):


(7) $$Entropy_{i}=\sum_{j=1}^{N}{d_{ij}}^{2}log({d_{ij}}^{2}),i=1,2,3,...,l$$

- **Non-linear feature set:** In non-linear feature set, non-linear parameters were calculated using Poincare plots of EEG time series. Poincare plot measures provide the non-linear dynamics that are embedded in MI-EEG signals. These measures were adopted and computed in this study considering their clinical ability demonstrated in similar studies (Isler, 2009; Narin et al., 2014). A Poincare plot is a simple 2-dimensional graph where each EEG sample (*x*_*i*_) is located on the x-axis and the next EEG sample (*x*_*i*+*lag*_) is located on the y-axis. After the indication of *x*_*i*_ and *x*_*i*+*lag*_ intervals, Poincare plots were obtained for each EEG signal. Thereafter, an ideal ellipse was applied to the graph of the Poincare plot, and the standard deviation of the distance of the points on these plots denoting the width (*SD*1) and length (*SD*2) of the ellipse (Brennan et al., 2001) were evaluated. The mathematical formulation of these measures is detailed as follows (Isler, 2009):

(8) $$\begin{array}{l} x_{i}=\left(x_{0},x_{1},...,x_{N-m}\right) \end{array}$$

(9) $$\begin{array}{l} x_{i+lag}=\left(x_{m},x_{m+1},...,x_{N}\right) \end{array}$$

(10) $$\begin{array}{l} x_{a}=\frac{x_{i+lag}-x_{i}}{\sqrt{2}}\\ x_{b}=\frac{x_{i+lag}+x_{i}}{\sqrt{2}} \end{array}$$

(11) $$\begin{array}{l} SD_{1}=SD\left(x_{a}\right)\\ SD_{2}=SD\left(x_{b}\right) \end{array}$$

In the Equations 8, 9, the EEG sample data and its next interval EEG data are indicated as *x*_*i*_ and *x*_*i*+*lag*_, respectively. *SD*_1_ and *SD*_2_ measures were evaluated using defined EEG intervals based on the Equations 10, 11. The standard deviation of the defined time interval vectors is denoted as *SD* in Equation 11. Also, the selected intervals were defined based on the *m*-lagged Poincare plot measurements. *lag=m* was defined, and m was identified as 1 and 9 for the interval-defining process. The measures were also calculated for the *lag=9* condition due to its positive outcomes from our previous study (Degirmenci et al., 2022a). The designed non-linear feature set, which *lag=9* condition provided a relevant and effective feature set for MI-EEG signals (Degirmenci et al., 2022a). Therefore, (*SD*1) and (*SD*2) measures were calculated for each *lag=m* condition. Additionally, the products (*SD*_1_*SD*_2_) and the rates (*SD*_1_/*SD*_2_) were calculated to examine the relations of (*SD*1) and (*SD*2). Four Poincare plot measures were calculated for each *lag=m* condition. In the non-linear feature set, a total of 8 Poincare plot measures were calculated from each EEG sample for two different *lag=m* conditions.

### 2.3 Statistical significance-based feature selection

Feature selection reduces the computational load by selecting effective and relevant features for classification (Narin et al., 2014). Selection of these features and application of them for classification improve the classification performances (Yesilkaya et al., 2023). There are several feature selection methods such as recursive feature selection (Al Ajrawi et al., 2024), LASSO regression (Huang et al., 2024; Muthukrishnan and Rohini, 2016), Correlation-based Feature Selection (CFS) (Kabir et al., 2023), Maximum Relevance Minimum Redundancy (MRMR) (Kabir et al., 2023), statistical significance-based feature selection (Bulut et al., 2022; Degirmenci et al., 2022b, 2023), and Genetic Algorithm (GA) (Ramos et al., 2016) for MI task classification in BCI research area. In this study, we preferred statistical significance-based feature selection methods since it is an easy-to-use method and its effectiveness has been proven in previous studies (Degirmenci et al., 2022b, 2023).

In this study, a total of four different feature sets were obtained, and the statistical significance-based feature selection process was performed for each of them separately. Additionally, motor imagery EEG signal classification was performed for both binary-class and multi-class extremity movement task classifications in this study. Thus, two different statistical significance based feature selection methods were employed to determine relevant and discriminative features. These methods are the independent *t*-test and one-way ANalysis Of VAriance (ANOVA), which are used for binary-class and multi-class extremity movement classifications, respectively. The class number of preferred classifications has an effect on the selection of the types of proposed feature selection method. The independent *t*-test was applied in feature selection of binary extremity movement classification to determine the relevant features of all provided feature sets. This method is mostly preferred to appear significance of differences between measures of two definite groups (Degirmenci et al., 2023; Narin et al., 2014). On the other hand, the ANOVA test, which is commonly utilized to show whether there is a difference between the means in states where there are two or more groups, was applied in multi-class extremity movement classification to determine the relevant features. Using these methods, *p*-values, which indicate the statistical significance of features, were calculated first. Then, the statistically significant features were determined considering the statistical significance level (α) equal to 0.05 in this study. Therefore, the effect of the independent *t*-test and ANOVA test was investigated on four different feature sets. To investigate and prove the effectiveness of these tests, the results of classifications performed using all extracted features and selected statistically significant features are compared.

### 2.4 Classification

In this study, EEG features in different feature sets have been classified by utilizing nine well-known classification algorithms. The selected classifiers and interrelated algorithms were applied by using the Classification Learner Toolbox, which is an element of Statistics and Machine Learning Toolbox available in the Matlab software package (Matlab, 2023). The technical information about corresponding machine learning algorithms is described below:

- **Decision tree:** Decision Tree (DT) is a type of common machine learning algorithm that creates and runs over structures consisting of root nodes, child nodes (e.g., leaf nodes), and branches (i.e., edges). The name of the classifier was inspired by its tree-like structure. It is a fast classification method and separates the data into various subgroups. In its structure, a feature is represented with each internal node of the tree; the feature combinations that result in classifications are indicated as branches of the tree, and class labels are indicated as leaves of the tree. The class of samples is predicted in the decision tree structure by evaluating from root to leaf (Tzallas et al., 2009; Sharma et al., 2022). The decision tree classifiers named the fine, medium, and coarse algorithms were employed in the classification process of this study.
- **Discriminant analysis:** The Discriminant Analysis (DA) classifier is one of the pattern recognition methods, and its main objective is to correctly separate the independent variables in the data into homogeneous groups (Chakrabarti et al., 2003). In this study, for the purpose of classification, linear and Quadratic Discriminant Analysis (QDA) algorithms were included in the study design. Among these classifiers, the LDA algorithm defines the group elements and estimates the probability that each element belongs to different groups. Then, the sample is assigned to the group with the highest probability result. It supposes that the predictors have a Gaussian distribution and are normally distributed. It also creates a linear discrimination function that assumes that different classes have class-specific elements and equal variance/covariance. Contrary to the LDA algorithm's assumption, variance/covariance equality is not accepted in the Quadratic Discriminant Analysis algorithm. In this algorithm, the covariance matrix may be different for each class category. Hence, it constructs the discrimination function to be quadratic (Lotte et al., 2018; Hart et al., 2000).
- **Logistic regression:** Logistic Regression (LR) is a commonly applied machine learning algorithm for binary classification. The fundamental process of this algorithm is based on representative of the probability of an event. Binary classification results are generated as outputs. In the technique of this algorithm, the logistic function, which is also known as the sigmoid function, is adapted to the corresponding data using probability (Tzallas et al., 2009). It maps the data points regarding a line, and all log-odds values are determined. These values are expressed as inputs and transformed to probability values. These calculated values are evaluated as the algorithm's outputs. Therefore, this input-output conversion is important for fitting the sigmoid function. In the classification process of this algorithm, the various line rotations are defined and evaluated via calculating, logging, and summing conditional probabilities for all steps. Then, the best sigmoid function that provided the maximum probability is calculated (Alkan et al., 2005).
- **Naive Bayes:** The Naive Bayes (NB) algorithm is a statistical classification approach that uses the Bayes Theorem on probability and variables' independence and normal state (Miao et al., 2017; Hart et al., 2000). Hence, all features provide the same effect value on the prediction process. The classification process is performed by employing the sample's likelihood of belonging to each class in the feature set. The class that provides the highest probability of membership is defined as the datum's predicted class. The Gaussian and kernel NB classifiers were incorporated and evaluated in this study.
- **Support Vector Machine:** The SVM algorithm, which was proposed by Vapnik (1999), is one of the well-known machine learning algorithms. It can be used in both classification and regression processes (Hart et al., 2000). It generates a model finding decision boundaries defined by a hyperplane to separate the data into categories based on the geometric characteristics of the data set. The optimum hyperplane that will best separate this data in space is selected to provide more accurate classification performance. The data is assigned as an element of a different class dependent upon which side of the hyperplane it is located on Bascil et al. (2016). In this study, various types of this algorithm, such as linear, quadratic, cubic, fine Gaussian, medium Gaussian, and coarse Gaussian, were adopted and run.
- **k-Nearest Neighbor:** The k-Nearest Neighbor (kNN) is a non-parametric machine learning algorithm, and it is computed in both classification and regression studies. It is a distance-based learning model and predicts the datum's class by evaluating the distance of the implemented sample to all k neighbors and assigning them as the one with the most prevalent neighbors (Tzallas et al., 2009; Isler, 2009). Various distance calculation methods are available (Hart et al., 2000). In this study, fine, medium, coarse, cubic, cosine, and weighted algorithms among the kNN classifiers are selected as classifiers. The Euclidean distance measurement method is one of the most computed distance calculation methods, and it is implemented for employing fine, medium, coarse, and weighted kNN algorithms (Hart et al., 2000; Isler, 2009). In cubic and cosine kNN algorithms, cubic and cosine distance measurement methods were utilized, respectively.
- **Ensemble learning:** As the name implies, it is an ensemble of classifier algorithms. Basically, different classifiers are brought together as a combination to form a single classifier algorithm. Ensemble methods, rather than the individual classifiers that make them up, generally provide far more accurate results thanks to different properties of classifiers such as reduction of variance (bagging), reduction of bias (boosting), and improvement predictions eliminating the over-fitting problem. Ensemble methods assume that a single prediction algorithm may not obtain precise and accurate classification results owing to some problems such as possible noise, overlapping data distributions, and outliers in the data (Khare et al., 2022; Matlab, 2023). Therefore, this method assumes that there is no single classifier that computes best for every classification study (Sayilgan et al., 2021b). Ensemble Learning (EL) algorithms have been frequently employed in recent research for the classification of different biomedical signals (Sayilgan et al., 2021b,a, 2022, 2020, 2019; Degirmenci et al., 2022a,b). In this study, Boosted, Bagged, Subspace Discriminant, Subspace k-NN, and RUSBoosted Trees algorithms are adopted.
- **Neural networks:** Neural networks (NN) classification algorithms are typically coined to have good predictive accuracy and can be used for multi-class classifications as well as binary classifications. Compared to other machine learning algorithms, the training process is longer due to the number of layers in their structure and many other parameters (Narin and Isler, 2021). NNs' architectural elements are layers of nodes, namely, the input layer, fully connected hidden layers, and output layer. Essentially, NNs' NNs differ by the number of fully connected hidden layers between the input and output layers, which affects the complexity of classifiers. Architectural complexity of NNs increases with the size and number of fully connected layers (Narin and Isler, 2021). The first fully connected layer of the neural network has a connection from the network input (predictor data), and each subsequent layer has a connection from the previous layer. Each fully connected layer multiplies the input data with a weight matrix and then adds a bias vector. An activation function follows each fully connected layer. The final fully connected layer and the next softmax activation function generate the output of the network, i.e., classification scores (next probabilities) and prediction labels (Narin and Isler, 2021; Richard and Lippmann, 1991; Pan et al., 2012). In a NN, to reach the optimum number of fully connected layers, as Geoffrey Hinton recommends, is to add layers until the model starts to overfit the training set (LeCun et al., 2015; Srivastava et al., 2014). In this study, narrow, medium, wide, bi-layered, and tri-layered NN algorithms were run for the classification process to investigate the effect of the size of the fully connected layers.
- **Kernel Approximation:** Kernel Approximation (KA) classifiers can be used to carry out non-linear classification of data containing many samples (Lei et al., 2019; Maji et al., 2008). In large datasets, KA classifiers tend to train and predict faster than SVM classifiers with Gaussian kernels (Maji et al., 2008). Gaussian kernel classification models map predictors in a low-dimensional space to a high-dimensional space and then generate a linear model to transform predictors in a high-dimensional space (Lei et al., 2019; Maji et al., 2008). In this study, support vector machine and logistic regression KA classifiers were employed for the purposes of classification.

### 2.5 Performance evaluation metrics

In this study, a 5-fold cross-validation method was performed to calculate the performance of the classification process by defining train, test, and validation data. In the evaluation of classification results, the reel labels of EEG segments were compared to those predicted by the classification algorithms. The MI classification results of machine learning algorithms consist of True Positive (TP), True Negative (TN), False Positive (FP), and False Negative (FN) in the binary and multiple classifications. Using these values, the statistical measure of Accuracy (ACC), which is the number of correctly predicted segments, is utilized for the evaluation of proposed methods. The mathematical formula for the accuracy performance metric is given in Equation 12 (Hart et al., 2000; Degirmenci et al., 2024).


(12) $$\begin{array}{l} ACC=\frac{TP+TN}{TP+FN+TN+FP} \end{array}$$

## 3 Results and discussion

In this study, the binary-class and multi-class classification problems of MI-EEG signals were analyzed with six different feature sets, a statistical significance-based feature selection method, and nine different classification algorithms. Firstly, four different feature sets were extracted from 22-channel EEG signals using time-domain, frequency-domain, time-frequency domain, and non-linear domain. Additionally, two different combinations of these feature sets were created. In addition to the investigation of the effects of different feature sets, the effects of the statistical significance-based feature selection method were investigated for extremity movement task classification. To show the effectiveness of this feature selection method, a classification process was also performed on EEG features themselves without any feature selection process. Afterward, all extracted feature sets and their reduced feature sets after the feature selection process was applied were evaluated with various machine learning algorithms separately.

Table 1 represents the list of all extracted feature types from four different domains and their abbreviations. Additionally, sizes of all extracted feature sets and statistical significance-based selected feature sets are represented in Table 2. The multi-class extremity movement task classification performances of all feature sets extracted through our suggested feature domains with ANOVA-based feature selection and various classifiers are given in Tables 3, 4. The boldface numbers in the tables represent the highest classification performance for that feature set. According to Table 3, the highest accuracy value (47.08%) was achieved using the non-linear feature set and support vector machine algorithm. However, the lowest classification performance was obtained using the time-frequency domain feature set. To investigate the advantages of different combinations of feature sets, the performances for combinations of feature sets are given in Table 4. The classification that was performed using the second combination feature set (TD+FD+WT+P) and ANOVA-based feature selection method improved the classification performance. Using this feature set, the highest accuracy value of 47.36% was achieved. Note here that the TD+FD+WT+P+ANOVA feature set with the EL algorithm provided the highest classification performance among all other approaches. The EL classifier gave the highest accuracy for almost all subjects for the multi-class extremity movement task classification process.

**Table 1:** The list of all time-domain, frequency-domain, time-frequency domain, and non-linear features.

**The code and name of all features**			
**T1**	Minumum value	**F1,W1**	Energy of delta band
**T2**	Maximum value	**F2,W2**	Variance of delta band
**T3**	Mean	**F3,W3**	Entropy of delta band
**T4**	Standard deviation value	**F4,W4**	Energy of theta band
**T5**	Integrated EEG value	**F5,W5**	Variance of theta band
**T6**	Mean absolute value	**F6,W6**	Entropy of theta band
**T7**	Simple square integral	**F7,W7**	Energy of alpha band
**T8**	Variance	**F8,W8**	Variance of alpha band
**T9**	Root mean square	**F9,W9**	Entropy of alpha band
**T10**	Waveform length	**F10,W10**	Energy of beta band
**T11**	Average amplitude change value	**F11,W11**	Variance of beta band
**T12**	Absolute difference in standard deviation	**F12,W12**	Entropy of beta band
**T13**	Kurtosis	**F13,W13**	Energy of gamma band
**T14**	Skewness	**F14,W14**	Variance of gamma band
**T15**	Hjorth parameters (Activity)	**F15,W15**	Entropy of gamma band
**T16**	Hjorth parameters (Mobility)	**P1**	SD1 where lag=1
**T17**	Hjorth parameters (Complexity)	**P2**	SD2 where lag=1
**T18**	Signal range	**P3**	SD1SD2 where lag=1
**T19**	First inter-quartile value (Q1)	**P4**	SD1/SD2 where lag=1
**T20**	Second inter-quartile value (Q2)	**P5**	SD1 where lag=9
**T21**	Third inter-quartile value (Q3)	**P6**	SD2 where lag=9
**T22**	Mode value	**P7**	SD1SD2 where lag=9
**T23**	Zero-crossing value	**P3**	SD1/SD2 where lag=9
**T24**	Slope-change value		

**Table 2:** Sizes of all feature sets and statistically significance-based selected feature sets applied for multi-class and binary-class extremity movement task classifications.

**Feature sets**	**Feature sets' dimension in multi-class classification**		**Feature sets' dimension in binary-class classification**	
	**All features**	**ANOVA selected features**	**All features**	***T*** **-test selected features**
	**(sample size x number of features)**			
TD	(2,592 × 528)	(2,592 × 345)	(1,296 × 528)	(1,296 × 44)
FD	(2,592 × 330)	(2,592 × 102)	(1,296 × 330)	(1,296 × 28)
WT	(2,592 × 330)	(2,592 × 104)	(1,296 × 330)	(1,296 × 13)
P	(2,592 × 176)	(2,592 × 61)	(1,296 × 176)	(1,296 × 6)
TD+FD+WT	(2,592 × 1,188)	(2,592 × 551)	(1,296 × 1,188)	(1,296 × 85)
TD+FD+WT+P	(2,592 × 1,364)	(2,592 × 612)	(1,296 × 1,364)	(1,296 × 91)

**Table 3:** Performance evaluation of all feature sets were tested for multi-class extremity movement task classification.

	**Feature sets**						
**Classifiers**	**Time-domain feature set**		**Frequency-domain feature set**		**Time-frequency domain feature set**		**Non-linear feature set**
	**TD**	**TD+ ANOVA**	**FD**	**FD+ ANOVA**	**WT**	**WT+ ANOVA**	**P (all lags)**
Decision Tree	31.00	31.10	31.40	31.44	28.32	28.74	31.90
Discriminant Analysis	41.90	**44.00**	34.41	37.89	N/A	25.42	40.20
Naive Bayes	29.40	29.40	28.59	29.09	28.20	28.16	28.30
Support Vector Machine	40.28	43.12	33.14	37.69	24.81	25.73	**47.08**
k-Nearest Neighbors	32.30	33.40	29.28	29.28	24.81	25.54	32.30
Ensemble Learning	**44.38**	43.91	**35.76**	**38.46**	**28.63**	**34.34**	46.06
Neural Networks	39.89	40.86	33.68	36.38	25.00	25.62	45.18
Kernel Approximation	32.48	31.87	32.18	30.94	24.81	25.81	30.63

The maximum component accuracies are shown in boldface for each feature set.

**Table 4:** Performance evaluation of combination feature sets was tested for multi-class extremity movement task classification.

	**Combination feature sets**			
**Classifiers**	**First combination feature set**		**Second combination feature set**	
	**TD+FD+WT**	**TD+FD+WT+ANOVA**	**TD+FD+WT+P**	**TD+FD+WT+P+ANOVA**
Decision Tree	34.38	34.34	34.50	34.50
Discriminant Analysis	N/A	25.96	N/A	27.31
Naive Bayes	29.09	29.51	27.90	29.43
Support Vector Machine	24.81	26.54	25.00	29.43
k-Nearest Neighbors	24.81	25.62	24.90	26.21
Ensemble Learning	**35.73**	**44.33**	**35.60**	**47.36**
Neural Networks	25.00	26.54	24.90	27.55
Kernel Approximation	24.81	25.46	24.90	25.89

The maximum component accuracies are shown in boldface for each feature set.

In this study, the detailed feature and channel analysis were implemented to analysis. The effects of different features and channels on classification performance were investigated by analyzing ANOVA-selected statistically significant features. In this direction, channel-based ANOVA-selected feature distribution maps were obtained to determine whether there was a distribution density in certain features or channels or not. If there were a certain statistically significant feature distribution density, its effect on performance after applying ANOVA-based feature selection was investigated by examining the feature distribution maps and classification results obtained. Channel-based ANOVA-selected statistically significant feature distributions for time-domain, frequency-domain, time-frequency domain, and non-linear domain are given in Tables 5–8.

**Table 5:** Channel-based ANOVA-selected statistically significant feature distribution among 22 EEG channels for multi-class extremity movement classification in the time-domain feature set.

	**EEG channels**																						
**F**	**1**	**2**	**3**	**4**	**5**	**6**	**7**	**8**	**9**	**10**	**11**	**12**	**13**	**14**	**15**	**16**	**17**	**18**	**19**	**20**	**21**	**22**	**T**
T1	✓	✓	✓	✓				✓	✓	✓	✓	✓		✓	✓	✓	✓	✓	✓	✓	✓	✓	18
T2		✓					✓	✓			✓	✓	✓	✓	✓	✓	✓	✓	✓	✓	✓	✓	15
T3	✓	✓	✓	✓	✓	✓	✓	✓	✓	✓	✓	✓	✓	✓	✓	✓	✓	✓	✓	✓	✓	✓	22
T4	✓	✓	✓	✓	✓	✓	✓	✓	✓	✓	✓	✓	✓	✓	✓	✓	✓	✓	✓	✓	✓	✓	22
T5	✓	✓	✓	✓	✓	✓	✓	✓	✓	✓	✓	✓	✓	✓	✓	✓	✓	✓	✓	✓	✓	✓	22
T6	✓	✓	✓	✓	✓	✓	✓	✓	✓	✓	✓	✓	✓	✓	✓	✓	✓	✓	✓	✓	✓	✓	22
T7	✓	✓	✓	✓	✓		✓	✓	✓	✓	✓	✓		✓	✓	✓	✓	✓	✓	✓	✓	✓	20
T8	✓	✓	✓	✓	✓		✓	✓	✓	✓	✓	✓		✓	✓	✓	✓	✓	✓	✓	✓	✓	20
T9	✓	✓	✓	✓	✓	✓	✓	✓	✓	✓	✓	✓	✓	✓	✓	✓	✓	✓	✓	✓	✓	✓	22
T10																							0
T11																							0
T12																							0
T13																							0
T14														✓				✓	✓	✓			4
T15	✓	✓	✓	✓	✓		✓	✓	✓	✓	✓	✓		✓	✓	✓	✓	✓	✓	✓	✓	✓	20
T16							✓	✓						✓	✓	✓	✓	✓	✓	✓	✓	✓	11
T17	✓	✓					✓	✓						✓	✓	✓			✓	✓	✓	✓	11
T18		✓	✓				✓	✓	✓	✓	✓	✓		✓	✓	✓	✓	✓	✓	✓	✓	✓	17
T19	✓	✓	✓	✓	✓	✓	✓	✓	✓	✓	✓	✓	✓	✓	✓	✓	✓	✓	✓	✓	✓	✓	22
T20	✓	✓	✓	✓	✓	✓	✓	✓	✓	✓	✓	✓	✓	✓	✓	✓	✓	✓	✓	✓	✓	✓	22
T21	✓	✓	✓	✓	✓	✓	✓	✓	✓	✓	✓	✓	✓	✓	✓	✓	✓	✓	✓	✓	✓	✓	22
T22		✓																					0
T23							✓	✓						✓	✓	✓		✓	✓	✓	✓	✓	10
T24	✓	✓	✓	✓	✓	✓	✓	✓	✓	✓	✓	✓	✓	✓	✓	✓	✓	✓	✓	✓	✓	✓	22
T	14	16	14	13	11	9	17	18	14	14	15	15	10	19	18	18	16	18	19	19	18	18	345

**Table 6:** Channel-based ANOVA-selected statistically significant feature distribution among 22 EEG channels for multi-class extremity movement classification in frequency-domain feature set.

	**EEG channels**																						
**F**	**1**	**2**	**3**	**4**	**5**	**6**	**7**	**8**	**9**	**10**	**11**	**12**	**13**	**14**	**15**	**16**	**17**	**18**	**19**	**20**	**21**	**22**	**T**
F1																							0
F2																							0
F3																							0
F4									✓	✓	✓				✓	✓	✓		✓	✓	✓	✓	10
F5										✓						✓				✓	✓	✓	5
F6		✓					✓															✓	3
F7	✓	✓	✓	✓	✓	✓	✓	✓	✓	✓	✓	✓	✓	✓	✓	✓	✓	✓	✓	✓	✓	✓	22
F8	✓	✓	✓	✓	✓	✓	✓	✓	✓	✓	✓	✓	✓	✓	✓	✓	✓	✓	✓	✓	✓	✓	22
F9												✓	✓					✓				✓	4
F10		✓	✓	✓	✓			✓	✓	✓	✓	✓		✓	✓	✓	✓	✓	✓	✓	✓	✓	18
F11			✓	✓	✓			✓	✓	✓	✓	✓		✓	✓	✓	✓	✓	✓	✓	✓	✓	18
F12																							0
F13																							0
F14																							0
F15																		✓					1
T	2	4	4	4	4	2	3	4	5	6	5	5	3	4	5	6	5	6	5	6	6	8	102

**Table 7:** Channel-based ANOVA-selected statistically significant feature distribution among 22 EEG channels for multi-class extremity movement classification in the time-frequency domain feature set.

	**EEG channels**																						
**F**	**1**	**2**	**3**	**4**	**5**	**6**	**7**	**8**	**9**	**10**	**11**	**12**	**13**	**14**	**15**	**16**	**17**	**18**	**19**	**20**	**21**	**22**	**T**
W1														✓					✓	✓	✓	✓	5
W2														✓					✓	✓	✓	✓	5
W3																						✓	1
W4	✓	✓	✓	✓	✓	✓	✓	✓	✓	✓	✓	✓	✓	✓	✓	✓	✓	✓	✓	✓	✓	✓	22
W5	✓	✓	✓	✓	✓	✓	✓	✓	✓	✓	✓	✓	✓	✓	✓	✓	✓	✓	✓	✓	✓	✓	22
W6																							0
W7	✓	✓	✓	✓	✓	✓	✓	✓	✓	✓	✓	✓	✓	✓	✓	✓	✓	✓	✓	✓	✓	✓	22
W8	✓	✓	✓	✓	✓	✓	✓	✓	✓	✓	✓	✓	✓	✓	✓	✓	✓	✓	✓	✓	✓	✓	22
W9																			✓				1
W10																							0
W11																							0
W12																							0
W13																							0
W14																							0
W15		✓					✓					✓								✓			4
T	4	5	4	4	4	4	5	4	4	4	4	5	4	6	4	4	4	4	7	7	6	7	104

**Table 8:** Channel-based ANOVA-selected statistically significant feature distribution among 22 EEG channels for multi-class extremity movement classification in non-linear feature set.

	**EEG channels**																						
**F**	**1**	**2**	**3**	**4**	**5**	**6**	**7**	**8**	**9**	**10**	**11**	**12**	**13**	**14**	**15**	**16**	**17**	**18**	**19**	**20**	**21**	**22**	**T**
P1	✓								✓			✓	✓			✓							5
P2					✓		✓	✓	✓		✓	✓								✓			7
P3	✓			✓	✓			✓			✓					✓			✓	✓			8
P4	✓			✓								✓			✓	✓			✓				6
P5		✓			✓	✓		✓	✓	✓		✓					✓			✓	✓		10
P6	✓	✓			✓			✓					✓		✓	✓	✓		✓	✓			10
P7	✓								✓			✓	✓		✓	✓			✓				7
P8					✓			✓	✓		✓	✓			✓				✓	✓			8
T	5	2	0	2	5	1	1	5	5	1	3	6	3	0	4	5	2	0	5	5	1	0	61

A total of 345 statistically significant time-domain features were evaluated on time-domain. According to Table 5, it was observed that the ANOVA-based feature selection process concentrated on 19 out of 24 time domain features and made almost no selection from 5 feature types (T10, T11, T12, T13, and T22). Additionally, the effects of channels were investigated on ANOVA-based feature selection. Among 22 EEG channels, statistically significant features were selected from almost all channels and feature selection process were not focused on certain channels. In order to investigate the effectiveness of the feature selection process in the time domain feature set, the classification performances were compared when all features and statistically significant time domain features were used (presented in Table 3). ANOVA-selected features from certain feature types and all channels improved the classification performance in 5 classifiers for time-domain based classifications.

In frequency domain, a total of 102 statistically significant frequency-domain features were selected. Channel-based statistically significant frequency-domain feature distribution is represented in Table 6. According to Table 6, it was observed that the ANOVA-based feature selection process focused on 6 out of 15 frequency-domain features and made no selection from 7 feature types (F1, F2, F4, F12, F13, F14 and F15). The energy and variance values of theta, alpha, and beta frequency bands were mostly selected as significant frequency-domain features among all frequency-domain features. Note here that alpha and beta frequency bands were associated with motor activities (Nicolas-Alanso and Gomez-Gil, 2012). Additionally, the effects of channels were investigated on ANOVA-based feature selection. Among 22 EEG channels, statistically significant features were mostly selected from certain channels (10th, 16th, 18th, 20th, 21st, and 22nd EEG channels). Among these certain channels, the 10th EEG channel was determined to be an effective channel for MI task classification studies in the literature (Xu et al., 2019). In order to investigate the effectiveness of the feature selection process in the frequency-domain feature set, the classification performances were compared when all features and statistically significant frequency-domain features were used (presented in Table 3). ANOVA-selected features from certain feature types and frequency bands, which are related with motor activities and certain channels (especially 10th EEG channel) improved the classification performance in almost all classifiers (6 classifiers) for frequency-domain based classifications.

In the WT-based time-frequency domain, a total of 104 statistically significant time-frequency domain features were selected. Table 7 represents the channel-based statistically significant time-frequency domain feature distribution. According to the feature distribution map, it was observed that the ANOVA-based feature selection process focused on 6 out of 15 time-frequency domain features and made almost no selection from 8 feature types (W3, W6, W9, W10, W11, W12, W13, and W14). The energy and variance values of delta, theta, and alpha frequency bands were mostly selected as significant time-frequency domain features among all time-frequency domain features. Among 22 EEG channels, the statistically significant features were mostly selected from certain channels (12th, 14th, 19th, 20th, 21th, and 22th EEG channels), but also statistically significant features were selected from all 22 EEG channels. In order to investigate the effectiveness of the feature selection process in the time-frequency domain feature set, the classification performances were compared when all features and statistically significant time-frequency domain features were used (presented in Table 3). ANOVA-selected features from certain feature types (energy and variance), certain effective frequency bands (especially the alpha band), which are related to motor activities, and all EEG channels improved the classification performance in almost all classifiers (6 classifiers) for time-frequency domain-based classifications.

Finally, the ANOVA-selected significant non-linear features were analyzed for the TD+FD+WT+P combination feature set. The ANOVA-based feature selection process determined 61 significant non-linear features. In feature selection, ANOVA selected from all non-linear feature types and it did not focus on some certain feature types. On the other hand, the ANOVA test selected significant non-linear features from almost all channels, but the feature selections were mostly realized from some certain channels (5th, 8th, 9th, 12th, 16th, 19th, and 20th EEG channels). In literature, 8th and 12th EEG channels are assigned as significant and discriminative EEG channels for MI-based BCI design (Xu et al., 2019). When the effect of ANOVA-based feature selection was investigated in the second combination feature set (TD+FD+WT+P), it was observed that these selections from the non-linear feature set improved the classification performance in almost all classifiers (6 classifiers) (represented in Table 4). Therefore, the highest performance of multi-task classification (47.36%) was achieved with the Ensemble learning classification algorithm by adding statistically significant non-linear features to the ANOVA-selected first combination feature set (TD+FD+WT+ANOVA).

The binary-class extremity movement task classification performances of all feature sets extracted through our suggested feature domains with the independent *t*-test-based feature selection and various classifiers are given in Tables 9, 10. The boldface numbers in the tables represent the highest classification performance for that feature set. According to Table 9, the highest accuracy value (63.04%) was achieved using the non-linear feature set and SVM algorithm binary classification. However, the lowest classification success was obtained using the time-frequency domain feature set. The performance evaluation of combination feature sets for binary extremity movement task classification is given in Table 10. The classification, which was performed using the first combination feature set (TD+FD+WT) and *t*-test-based feature selection method, achieved the highest accuracy value of 62.96% among two different combination feature sets. The results revealed that the combination of different feature sets did not improve classification performance for binary classification. A non-linear feature set provided the highest classification performance. EL classifier gave the highest accuracy for almost all subjects for the binary-class extremity movement task classification process.

**Table 9:** Performance evaluation of all feature sets was tested for binary-class extremity movement task classification.

	**Feature Sets**						
**Classifiers**	**Time-domain feature set**		**Frequency-domain feature set**		**Time-frequency domain feature set**		**Non-linear feature set**
	**TD**	**TD+** ***T*****-test**	**FD**	**FD+** ***T*****-test**	**WT**	**WT+** ***T*****-test**	**P (all lags)**
Decision Tree	56.56	55.02	57.56	57.48	**52.70**	50.62	53.32
Discriminant Analysis	57.64	56.02	53.86	61.11	N/A	50.93	54.63
Logistic Regression	56.17	55.79	54.63	**61.34**	49.85	50.77	53.24
Naive Bayes	52.62	55.17	52.01	55.79	51.16	**54.71**	48.53
Support Vector Machine	59.57	56.64	55.63	59.03	N/A	50.93	**63.04**
k-Nearest Neighbors	53.24	54.32	52.01	54.78	49.85	50.69	53.16
Ensemble Learning	**61.26**	**57.72**	**60.03**	60.26	51.39	53.94	61.19
Neural Networks	58.72	53.01	56.48	57.18	N/A	50.54	60.57
Kernel Approximation	54.24	51.08	55.94	53.55	N/A	49.15	53.86

The maximum component accuracies are shown in boldface for each feature set.

**Table 10:** Performance evaluation of combination feature sets was tested for binary-class extremity movement task classification.

	**Combination feature sets**			
**Classifiers**	**First combination feature set**		**Second combination feature set**	
	**TD+FD+WT**	**TD+FD+WT+** ***T*** **-test**	**TD+FD+WT+P**	**TD+FD+WT+P+** ***T*** **-test**
Decision Tree	56.71	55.25	56.60	56.50
Discriminant Analysis	N/A	51.23	N/A	52.10
Logistic Regression	49.92	51.00	49.90	51.10
Naive Bayes	53.47	57.02	48.50	57.10
Support Vector Machine	N/A	51.16	N/A	51.40
k-Nearest Neighbors	49.85	50.31	49.80	50.80
Ensemble Learning	**58.10**	**62.96**	**57.30**	**61.86**
Neural Networks	N/A	50.85	N/A	50.54
Kernel Approximation	N/A	50.00	N/A	49.92

The maximum component accuracies are shown in boldface for each feature set.

The distribution of the independent *t*-test selected statistically significant features among 22 EEG channels was investigated for binary-class extremity movement task classification. Channel-based *t*-test selected significant time-domain, frequency-domain, time-frequency domain, and non-linear domain feature distributions are given in Tables 11–14, respectively. In the time domain, a total of 44 statistically significant features were selected using a *t*-test. The mean value and first interquartile value (Q1) were mostly selected features among 24 different time-domain feature types. The effect of the channels were investigated, and it was observed that significant features mostly selected some channels (6th, 11th, 12th, and 18th EEG channels) and no selection was made from some EEG channels. The analyses revealed that the *t*-test-based feature selection process, which focused on certain EEG channels and certain features such as mean value and Q1 in the time-domain feature set, did not improve the classification performance in almost majority of the classifiers (8 classifiers) (represented in Table 9).

**Table 11:** Channel-based *t*-test selected statistically significant feature distribution among 22 EEG channels for binary-class extremity movement classification in a time-domain feature set.

	**EEG channels**																						
**F**	**1**	**2**	**3**	**4**	**5**	**6**	**7**	**8**	**9**	**10**	**11**	**12**	**13**	**14**	**15**	**16**	**17**	**18**	**19**	**20**	**21**	**22**	**T**
T1																							0
T2				✓	✓	✓																	3
T3					✓	✓					✓	✓	✓			✓	✓	✓	✓	✓	✓	✓	12
T4																							0
T5																							0
T6																							0
T7																							0
T8																							0
T9																							0
T10																							0
T11																							0
T12																							0
T13				✓	✓						✓	✓											4
T14						✓							✓					✓					3
T15																							0
T16																							0
T17																							0
T18																							0
T19												✓	✓				✓	✓				✓	5
T20					✓	✓					✓	✓	✓		✓	✓	✓	✓	✓	✓	✓	✓	13
T21						✓																	1
T22																							0
T23																							0
T24												✓	✓					✓					3
T	0	0	0	2	4	5	0	0	0	0	3	5	5	0	1	2	3	5	2	2	2	3	44

The distribution of the *t*-test selected significant frequency-domain features were investigated in Table 12. The mostly selected features were energy, variance and entropy of the alpha band, and entropy of the theta band. According to literature studies, alpha and beta bands are mostly associated with motor activities (Nicolas-Alanso and Gomez-Gil, 2012). Among these bands, especially the alpha band reflects the changes during memory and brain function. Alpha-band rhythms are placed in the same frequency range as Mu rhythms, which are robustly associated with motor activities. The *t*-test-based feature selection method verified the literature focusing on the alpha frequency band. Among all EEG channels, more frequency features were determined as significant features from certain channels (12th and 18th). 12th EEG channel was determined as an effective EEG channel for MI-EEG signal analysis in the literature (Xu et al., 2019). Note here that these remarkable feature selections in the frequency-domain improved the classification performance in almost all classifiers (7 classifiers), as shown in Table 9.

**Table 12:** Channel-based *t*-test selected statistically significant feature distribution among 22 EEG channels for binary-class extremity movement classification in a frequency-domain feature set.

	**EEG channels**																						
**F**	**1**	**2**	**3**	**4**	**5**	**6**	**7**	**8**	**9**	**10**	**11**	**12**	**13**	**14**	**15**	**16**	**17**	**18**	**19**	**20**	**21**	**22**	**T**
F1																							0
F2																							0
F3																							0
F4																							0
F5																							0
F6					✓		✓	✓											✓	✓			5
F7	✓	✓										✓	✓	✓			✓	✓			✓		8
F8												✓	✓				✓	✓			✓		5
F9					✓	✓					✓	✓	✓					✓				✓	7
F10																							0
F11												✓						✓					2
F12																							0
F13																							0
F14																							0
F15																		✓					1
T	1	1	0	0	2	1	1	1	0	0	1	4	3	1	0	0	2	5	1	1	2	1	28

Table 13 represents the distribution of the statistically significant features in time-frequency domain. The *t*-test selected 13 different significant features, and the most selected features were entropy features in this domain. Entropy of delta, alpha, beta, and gamma bands were especially selected features with the application of the *t*-test. In addition to the frequency bands (alpha and beta activities) that are effective in the MI imaginary task in the literature (Nicolas-Alanso and Gomez-Gil, 2012), other frequency bands (delta and gamma activities) were also selected. Among 22 EEG channels, more significant features were selected from 2 EEG channels (4th and 20th EEG channels), which are not indicated as effective EEG channels for MI-EEG analysis in the literature. Contrary to the literature, *t*-test based feature selection improved the classification performance in half of the classifiers (4 classifiers) by selecting statistically significant features in different frequency bands and without focusing on effective channels in the literature (represented in Table 9).

**Table 13:** Channel-based *t*-test selected statistically significant feature distribution among 22 EEG channels for binary-class extremity movement classification in the time-frequency domain feature set.

	**EEG channels**																						
**F**	**1**	**2**	**3**	**4**	**5**	**6**	**7**	**8**	**9**	**10**	**11**	**12**	**13**	**14**	**15**	**16**	**17**	**18**	**19**	**20**	**21**	**22**	**T**
W1																							0
W2																							0
W3				✓																	✓	✓	3
W4																							0
W5																							0
W6		✓																			✓		2
W7																		✓					1
W8																							0
W9																							0
W10																							0
W11																							0
W12										✓				✓									2
W13																							0
W14																							0
W15				✓			✓					✓			✓					✓			5
T	0	1	0	2	0	0	1	0	0	1	0	1	0	1	1	0	0	1	0	1	2	1	13

Finally, the channel-based *t*-test selected significant non-linear feature distribution among 22 EEG channels and was investigated in Table 14. Of the total 176, 6 were determined to be significant features in this domain. The mostly selected feature type was *SD*1/*SD*2, where *lag* = 9. Note here that the fact that the *t*-test determined the extracted features for the lag = 9 case as statistically significant explains why we included them in the combination feature set. On the other hand, the 12th EEG channel provided statistically significant features during *t*-test application in the non-linear domain. This channel revealed its effectiveness for MI-EEG signal analysis in the literature (Xu et al., 2019). As a result, *t*-test based feature selection process improved classification performance (4 classifiers) by selecting certain non-linear features and 12th EEG channel (represented in Table 10).

**Table 14:** Channel-based *t*-test selected statistically significant feature distribution among 22 EEG channels for binary-class extremity movement classification in a non-linear feature set.

	**EEG channels**																						
**F**	**1**	**2**	**3**	**4**	**5**	**6**	**7**	**8**	**9**	**10**	**11**	**12**	**13**	**14**	**15**	**16**	**17**	**18**	**19**	**20**	**21**	**22**	**T**
P1																							0
P2																							0
P3																							0
P4																							0
P5																		✓					1
P6																							0
P7																							0
P8											✓	✓	✓				✓	✓					5
T	0	0	0	0	0	0	0	0	0	0	1	1	1	0	0	0	1	2	0	0	0	0	6

The main results and contributions of our extremity movement task classification study can be highlighted as follows:

- We propose different feature extraction methods considering different feature domains such as time-domain, frequency-domain, time-frequency domain, and non-linear domain.
- Among all proposed feature sets, the second combination feature set (TD+FD+WT+P) with ANOVA and non-linear feature set give the highest classification accuracies for multi-class and binary-class extremity movement task classifications, respectively. On the other hand, the lowest classification Results were generally obtained using a WT-based time-frequency feature set in both multi-class and binary-class cases.
- In all feature sets, the best classification results were mostly provided with the EL classifier among all classifiers.
- The classification results on all feature sets show that the statistical significance-based feature selection method generally improved classification performances in majority of the classifiers.
- According to the authors' knowledge, this is the first MI-EEG signal analysis study in which detailed feature and channel analysis have been implemented to identify effective and discriminative features and channels using statistically significant feature distribution maps.
- The feature and channel analyses we have performed have shown that features can be successfully selected from frequency bands, features, and channels whose effectiveness has been determined in the literature by our statistically significant based feature selection method. In addition, it has been observed that classifier performance can be increased by selecting statistically significant-based features from different channels and features other than those in the literature. The analyses performed in the study both support the literature and add new features and channels. Note here that, instead of accepting the effectiveness of some definite channels and features according to the literature and eliminating some of them, we can improve the classifier performance by performing detailed feature and channel analyses with feature selection based on statistical significance.

Table 15 shows the comparison of various multi-class and binary-class extremity movement task classification literature studies with the results of this study. The main contributions and drawbacks of these studies were analyzed considering the authors' proposed methods such as feature extraction, feature selection, and classification methods. According to Table 15, the majority of these studies include high computational complexity due to feature extraction (Sakhavi et al., 2015; Tabar and Halici, 2016; Dong et al., 2020; Amiri et al., 2024), feature selection (Molla et al., 2020; Jusas and Samuvel, 2019; Lu et al., 2020; Garcia-Laencina et al., 2014; Ma et al., 2018), and classification (Sakhavi et al., 2015; Ma et al., 2018; Nguyen et al., 2018; Dong et al., 2020; Amiri et al., 2024; Tabar and Halici, 2016; Djamal et al., 2017; Xu et al., 2019; Zhao et al., 2019) methods. Although the approaches suggested by the authors created a computational load on the BCI system, very high performance values could not be achieved in both types of classification cases. In some studies (Garcia-Laencina et al., 2014; Jusas and Samuvel, 2019; Amiri et al., 2024; Gaur et al., 2015; Djamal et al., 2017; Xu et al., 2019; Lu et al., 2020), although channel selection was applied and certain channels were selected for MI-EEG analysis, the performances still did not reach high values. Also, literature studies classifying binary and multiple MI tasks were interested in coding MI tasks by disregarding the brain's idle case (i.e., the state that the brain is not performing any mental tasks). This situation may result in an increase in the number of FPs and decrease the classification performances dramatically (Degirmenci et al., 2024). The study including the no mental task (NoMT) condition is available in the literature (Degirmenci et al., 2024). Since there was no EEG signal for NoMT condition in the dataset (BCI Competition IV Dataset IIa) we worked with, we could not include it and examine its effect, but the effectiveness of this situation can be tested with different datasets. On the other hand, to the best of the authors' knowledge, none of MI task classification studies, or even in the literature, have applied detailed feature and channel effectiveness research for MI task classification as our previous MI-EEG signals classification study (Degirmenci et al., 2023). In our previous study, we extracted MI-EEG features from different feature extraction domains and combined them. Then, the statistical significance-based feature selection method was applied to improve classifiers' performance. In that study, only the effectiveness of statistically significant features on the classification performance is investigated, regardless of which feature types or channels the statistically significant features are selected from. Our main goal in that study was to find the best classification performance and investigate the effect of feature selection based on statistical significance. For this reason, the study did not include feature and channel analysis. Therefore, this study (i) has computational advantages in terms of the methods which were applied in feature extraction, feature selection, and classification process, (ii) used all EEG channels and four different feature domains instead of conducting a study based solely on certain feature extraction methods and EEG channels, (iii) was also investigated the effectiveness of a wide range of classification algorithms not previously used in MI-EEG signal analysis in the literature (such as DT, EL, LR, and KA classifiers), and (iv) implemented the detailed feature and channel analysis using channel-based statistically significant feature distribution maps among 22 EEG channels for each extracted feature set.

**Table 15:** Comparison of various multi-class and binary-class extremity movement task classification studies with the results of the study.

**References**	**Dataset**	**C**	**c**	**F**	**Classifier**	**ACC**
Garcia-Laencina et al. (2014)	Five sets of EEG data (publicly available)	2	5	22	Linear discriminant analysis	77.30
Lindig-Leon and Bougrain (2015)	OpenVibe platform-based publicly available dataset	26	4	N/A	Linear discriminant analysis	51.67
Sakhavi et al. (2015)	BCI Competition IV Dataset IIa	22	4	8	Convolutional neural network	70.60
Ma et al. (2018)	Publicly available EEG Movement/Imagery Database (eegmmidb)	64	5	N/A	Recurrent neural networks	68.20
Nguyen et al. (2018)	BCI Competition IV Dataset IIa + Dataset IIIa	22	4	16	Fuzzy logic system	65.00
Jusas and Samuvel (2019)	BCI Competition IV Dataset IIa	8	4	46	Support vector machines
Lee et al. (2019)	Their own dataset	64	4	N/A	Linear discriminant analysis	58.20
Dong et al. (2020)	BCI Competition IV Dataset IIa	22	4	N/A	Relevance vector machine	74.39
Kato et al. (2020)	Open access MI dataset	21	5	189	Support vector machines	40.60
Amiri et al. (2024)	BCI Competition IV Dataset IIa	6	4	N/A	Convolutional neural network	72.01
**This study**	**BCI Competition IV Dataset IIa**	**22**	**4**	**612**	**Ensemble Learning**	**47.36**
Gaur et al. (2015)	BCI Competition IV Dataset IIa	3	2	N/A	Linear discriminant analysis	70.20
Tabar and Halici (2016)	BCI Competition IV Dataset IIb	22	2	N/A	Convolutional neural networks	77.60
Djamal et al. (2017)	Their own dataset	1	2	N/A	Learning vector qantization networks	70.00
Xu et al. (2019)	BCI Competition IV Dataset IIb	3	2	N/A	Convolutional neural network	74.20
Zhao et al. (2019)	BCI Competition IV Dataset IIb	22	2	N/A	Convolutional neural network	83.00
Lu et al. (2020)	BCI Competition IV Dataset IIb	2	2	N/A	Ensemble support vector learning	71.00
Molla et al. (2020)	BCI Competition IV Dataset IIb	22	2	15	Support vector machine	81.52
**This study**	**BCI Competition IV Dataset IIa**	**22**	**2**	**176**	**Ensemble Learning**	**63.04**

ACC is the highest accuracy achieved in the cited research and this study. In the table, F stands for “Number of features,” C stands for “Number of channels,” c stands for “Number of classes,” and ACC stands for “Accuracy.”

## 4 Conclusion

Despite the advances in both traditional machine learning (ML) and deep learning (DL) methods, there are several challenges in developing EEG-based BCI systems (Rashid et al., 2020). In traditional ML methods, the model's success largely depends on the features extracted from the raw EEG signals (Khademi et al., 2022). Extracting these features requires domain expertise and is time consuming. Moreover, the high-dimensional and noisy nature of EEG data limits scalability and generalizability. In contrast, DL methods, which are increasingly gaining popularity in EEG analysis in recent decades, have the potential to solve some of these problems by automatically learning complex features directly from the raw signal (Praveena et al., 2020) . DL models can eliminate temporal and spatial dependencies in EEG data more effectively than traditional ML methods. However, DL models require large amounts of data to learn complex patterns effectively. This represents a significant obstacle to the application of DL models to EEG-based studies. Moreover, in EEG analysis, traditional ML models are simpler and more interpretable, while DL models appear as completely “closed black boxes.” This lack of transparency is a major hurdle in the medical field, where clinicians need clear and explainable reasons for diagnostic decisions. Moreover, DL models require significant computational resources for both training and real-time inference, which is another major limitation for portable or real-time applications such as BCIs. In summary, EEG-based BCI studies attempt to overcome the ongoing challenges of feature extraction and selection in traditional ML, while DL attempts to overcome challenges related to data scarcity, model interpretability, and computational requirements.

In this study, we developed an automated signal processing study to design a MI-EEG based BCI system. First, various time-domain, frequency-domain, time-frequency, and non-linear domain features of EEG segments were obtained from their corresponding domains. Then, the impact of the statistical significance-based feature selection method was investigated on these four feature sets and their two different combination feature sets. The proposed feature selection method was analyzed by comparing all features separately and selecting statistically significant features in all feature sets separately. Finally, the classification was performed using 9 different machine learning methods and 5-fold cross-validation method. The applied methods were tested on two different classification cases (binary class and multi class extremity movement task classification). The methods proposed in this study were tested on the publicly available BCI Competition IV Dataset IIa dataset in order to validate its robustness, and compare its success with literature studies. The purpose of this study was not to find the best classifier but to determine the effective channels or features in MI EEG signals classification. In accordance with this purpose, the detailed feature and channel analysis was implemented in this study because the selection and application of channel selection, feature extraction, and feature selection methods are very important in BCI system design. Especially, the detailed feature and channel analysis was performed for the first time to analyze MI-EEG signals as well as various feature domains, and to the best of our knowledge, some classification algorithms were used to classify MI-EEG signals for the first time. The results showed that the highest accuracy rates of 47.36% and 63.04% were obtained when using the second combination feature set (TD+FD+WT+P) and the non-linear feature set on multi-class and binary-class classifications, respectively. The statistical significance-based feature selection method generally improved the classification performances in selecting significant and discriminative EEG features from different feature sets. According to experimental analysis, the nonlinear and second combination feature sets (TD+FD+WT+P) were found to be the feature sets with the best results. Among all the classification algorithms, the EL classifier was found to be the most successful machine learning method for all feature sets tested in this study. The feature and channel analyses performed in the study revealed that the distribution of statistically significant features across 22 EEG channels both support the literature by selecting certain frequency bands, features and channels that have been proven to be effective for MI-EEG signal classification and add new features and channels to them. Therefore, the classifier' performance can be improved by performing detailed feature and channel analysis using feature selection based on statistical significance. On the other hand, the methods used in this BCI design study have lower complexity than majority of MI-EEG signal classification approaches.

## Data Availability

Publicly available datasets were analyzed in this study. This data can be found here: http://www.bbci.de/competition/iv/desc_2a.pdf.
